# Unmasking Indolent Systemic Mastocytosis in Patients with Unexplained or Treatment-Refractory Osteoporosis: A Case Series with Diagnostic and Therapeutic Implications

**DOI:** 10.3390/biom16060821

**Published:** 2026-06-01

**Authors:** Lucia Jankovski, Rok Herman, Matej Rakusa, Peter Kopač, Mark Kačar, Matevž Škerget, Andrej Janež, Mojca Jensterle

**Affiliations:** 1 Department of Internal Medicine, University Medical Centre Ljubljana, 1000 Ljubljana, Slovenia; lucia.jankovski@gmail.com; 2 Department of Endocrinology, Diabetes and Metabolic Diseases, University Medical Centre Ljubljana, 1000 Ljubljana, Slovenia; 3 Faculty of Medicine, University of Ljubljana, 1000 Ljubljana, Slovenia; 4 Department of Health Sciences, Alma Mater Europaea University, Slovenska cesta 17, 2000 Maribor, Slovenia; 5 Division of Allergology, University Clinic of Respiratory and Allergic Diseases Golnik, 4204 Golnik, Slovenia; 6 Department of Haematology, University Medical Centre Ljubljana, 1000 Ljubljana, Slovenia

**Keywords:** indolent systemic mastocytosis, osteoporosis, fragility fracture, serum tryptase, KIT D816V, bone mineral density, bone turnover markers, zoledronic acid, avapritinib, secondary osteoporosis

## Abstract

Indolent systemic mastocytosis (ISM) is an under-recognised cause of secondary osteoporosis, and skeletal fragility may be the only presenting feature, delaying diagnosis. We describe four adults referred to a tertiary endocrinology service for unexplained osteoporosis or low-trauma fractures, in whom systemic mastocytosis (SM) was identified during work-up. All had elevated basal serum tryptase (41.4–87.0 µg/L), bone-marrow biopsy showing atypical mast cells and the *KIT* D816V variant; cutaneous lesions were absent in every case. Three patients fulfilled WHO 2022 criteria for ISM. The fourth had coexistent *JAK2* V617F-positive post-essential-thrombocythaemia myelofibrosis and was classified as SM with associated haematological neoplasm (SM-AHN); his mast cell clone (tryptase 43.7 µg/L; *KIT* D816V VAF 0.391%) behaved indolently and contributed clinically through osteoporosis alone, illustrating that an indolent mast cell component can be overlooked when a chronic myeloid neoplasm dominates the picture. Presentations ranged from an isolated low-energy L5 fracture in a 55-year-old man, to multiple vertebral compression fractures despite denosumab in a 71-year-old woman with primary hyperparathyroidism, to severe wasp-sting anaphylaxis in a 43-year-old man. After multidisciplinary review, all received intravenous zoledronic acid with vitamin D repletion; KIT-targeted therapy is under consideration in selected patients. Although causal inferences cannot be drawn from four retrospectively identified cases, the series illustrates how ISM may be missed in unexplained or treatment-refractory osteoporosis—particularly in younger men, those with prior severe anaphylaxis, and those fracturing on antiresorptive therapy—and supports combining basal serum tryptase with high-sensitivity peripheral-blood *KIT* D816V testing, in line with the WHO/ICC/AIM-ECNM 2022–2024 criteria. Prospective studies are needed.

## 1. Introduction

Mastocytosis comprises a heterogeneous group of disorders characterised by clonal expansion and tissue accumulation of mast cells (MCs), most often within the bone marrow (BM) and skin [1]. Its clinical spectrum is broad. In children, the disease is typically confined to the skin (cutaneous mastocytosis), whereas systemic mastocytosis (SM), defined by MC infiltration of at least one extracutaneous organ such as the BM, spleen, lymph nodes, gastrointestinal tract, or skeleton, predominates in adults [2]. Indolent systemic mastocytosis (ISM) is the most common adult subtype and produces a wide variety of MC-mediator-driven symptoms, including fatigue, pruritus, gastrointestinal complaints, severe anaphylaxis and skeletal fragility [2].

Skeletal involvement is one of the most prevalent systemic features of ISM. Osteoporosis is reported in approximately 20–38% of the patients, osteopenia in about 32% and osteosclerosis in up to 6%; a third of patients sustain fragility fractures during follow-up, and reduced bone mineral density (BMD) is not uncommon in younger adults [3,4,5,6,7,8,9]. The underlying pathophysiology is multifactorial: MC-derived mediators, among them histamine, tryptase, heparin and a range of cytokines, enhance osteoclastogenesis, suppress osteoblast activity and disrupt the equilibrium of bone remodelling, while neoplastic MC infiltration of the BM may produce focal lytic or sclerotic lesions [3,8]. Bone disease may therefore range from asymptomatic BMD loss to disabling fractures, and in a meaningful minority of patients, it is the first or only manifestation of ISM [2,10].

Because skeletal fragility can be the presenting feature of ISM, adults with unexplained low BMD or recurrent low-trauma fractures, particularly those with prior severe anaphylaxis, may warrant consideration of ISM in the differential diagnosis, with basal serum tryptase and, where indicated, peripheral-blood screening for the *KIT* D816V mutation [2,3]. Timely recognition is crucial, as effective therapies exist: among antiresorptives, zoledronic acid has the strongest evidence base, denosumab is an accepted alternative and data for teriparatide remain limited and potentially unfavourable [3,7,11,12]. Even so, skeletal events may continue despite adequate antiresorptive therapy, highlighting the need for a mechanism-based, multidisciplinary approach.

Several clinically important gaps persist. The majority of published series were identified through haematology or allergy services. In a metabolic bone clinic, rather than a haematology unit, patients’ primary presentation is skeletal fragility, without cutaneous lesions or MC-mediator symptoms. Coexistence of ISM with other conditions that independently cause bone loss, such as primary hyperparathyroidism or myeloproliferative neoplasms, is rarely described in detail.

Our group has previously summarised the molecular and therapeutic landscape of ISM-related bone disease in a narrative review without reporting individual patients [3]. The present work is conceived as a complementary clinical case series, restricted to four newly described patients and centred on the diagnostic and therapeutic implications that emerge from these specific cases. No patient, image or numerical result is shared between the two articles. The primary aim is to illustrate the pathway by which adults referred for unexplained or treatment-refractory osteoporosis came to be recognised as having systemic mastocytosis.

## 2. Case Presentation

The series comprises four consecutive patients (three men and one woman) in whom osteoporosis with fragility fractures led to a diagnosis of ISM in the last two years. The demographic, biochemical and densitometric findings are summarised in Table 1.

### 2.1. Case 1

A 55-year-old man was referred for evaluation of severe osteoporosis after developing back pain one week after stacking firewood one year prior to medical assessment. Magnetic resonance imaging showed a low-energy L5 vertebral fracture. There were no identifiable risk factors for secondary osteoporosis: no prior malignancy, nephrolithiasis, radiation exposure or glucocorticoid use; stable body weight; a regular mixed diet including dairy products; and no history of smoking or harmful alcohol use. His mother had osteoporosis.

Dual-energy X-ray absorptiometry (DXA) confirmed osteoporosis: lumbar spine T-score −4.0 (BMD 0.612 g/cm^2^); femoral neck T-score −2.1 (BMD 0.650 g/cm^2^); total hip T-score −1.3 (BMD 0.844 g/cm^2^). Calcium and phosphate homeostasis was intact, but 25-hydroxyvitamin D was below the recommended range. Bone turnover markers were in the low-normal range (CTX 0.092 µg/L; PINP 67.5 µg/L). During the work-up for secondary osteoporosis, basal serum tryptase was markedly elevated at 41.4 µg/L (reference range [RR] < 11.4 µg/L), prompting referral to haematology.

Bone-marrow (BM) biopsy demonstrated multifocal dense aggregates of atypical, spindle-shaped mast cells (MCs) with aberrant CD2/CD25 co-expression, and molecular testing confirmed the *KIT* D816V mutation; VAF was not assessed. There were no cutaneous lesions, no B-findings (BM MC infiltration < 30%; tryptase < 200 µg/L; no organomegaly or dysmyelopoiesis) and no C-findings. The findings fulfilled the WHO 2022 criteria for indolent systemic mastocytosis (ISM): one major criterion plus three minor criteria (atypical morphology, KIT D816V, basal tryptase > 20 µg/L).

Treatment was initiated with intravenous zoledronic acid 5 mg yearly together with cholecalciferol repletion. Over 13 months of joint haematology–endocrinology follow-up, the patient remained free of new fractures. Repeat basal serum tryptase was stable.

### 2.2. Case 2

A 39-year-old man with previously established *JAK2* V617F-positive post-essential-thrombocythaemia (post-ET) myelofibrosis—diagnosed ten years earlier after splanchnic venous thrombosis (portal, splenic and mesenteric veins) and treated with warfarin and ruxolitinib—was assessed for suspected secondary osteoporosis with pathological vertebral fractures. A remote childhood injury (left radius and ulna fractures at age 15 with refracture at age 26, when radiographs already suggested reduced bone mineral density) had not been further investigated at the time. He experienced several months of progressive back pain that worsened abruptly during a jump while playing basketball. He was a former smoker, had not received glucocorticoids and had no family history of metabolic bone disease.

Imaging showed an acute T12 vertebral compression fracture and an older L1 fracture, both managed conservatively; no antiresorptive therapy or vitamin D supplementation had previously been prescribed. DXA showed lumbar spine T-score −2.5 (BMD 0.843 g/cm^2^), femoral neck T-score −1.4 (BMD 0.733 g/cm^2^) and total hip T-score −0.6 (BMD 0.941 g/cm^2^). Vitamin D was insufficient. Bone turnover markers were within or near the normal range (CTX 0.274 µg/L; PINP 50.9 µg/L). Basal serum tryptase was elevated at 43.7 µg/L (RR < 11.4 µg/L), raising suspicion of concomitant mastocytosis.

Repeat BM biopsy fulfilled the major SM criterion (multifocal dense MC infiltrates) together with the minor criteria of atypical spindle-shaped morphology, aberrant CD2/CD25 expression and *KIT* D816V (variant allele frequency 0.391%). There were no cutaneous lesions and no B- or C-findings. Because these findings coexisted with the previously established *JAK2* V617F-positive post-ET myelofibrosis, the final classification under the WHO 5th edition (2022) and ICC 2022 criteria is systemic mastocytosis with an associated haematological neoplasm (SM-AHN)—an advanced-SM subtype distinct from ISM. The MC clone itself, characterised by a moderately elevated tryptase (43.7 µg/L) and a low *KIT* D816V VAF (0.391%), behaved indolently and contributed clinically through osteoporosis alone.

After haematology consultation, intravenous zoledronic acid was initiated together with vitamin D repletion. Over 16 months of combined endocrinology–haematology follow-up, no additional fractures occurred; repeat DXA at 12 months showed stable BMD, and serial basal tryptase remained at the same level. The patient continues under combined endocrinology and haematology care; KIT-targeted therapy or allogeneic haematopoietic stem-cell transplantation will be considered if systemic manifestations progress.

### 2.3. Case 3

A 43-year-old man had a history of T12 and L3 vertebral fractures following a motor-vehicle collision 10 years earlier, and a subsequent spontaneous rib fracture after an abrupt movement 4 years later. Reduced BMD, suspected during the initial trauma work-up, was confirmed by DXA. He had previously experienced a grade IV anaphylactic reaction to a wasp sting, after which he completed Hymenoptera venom immunotherapy; basal serum tryptase at the time of the index anaphylaxis had not been measured.

**Figure 1:**
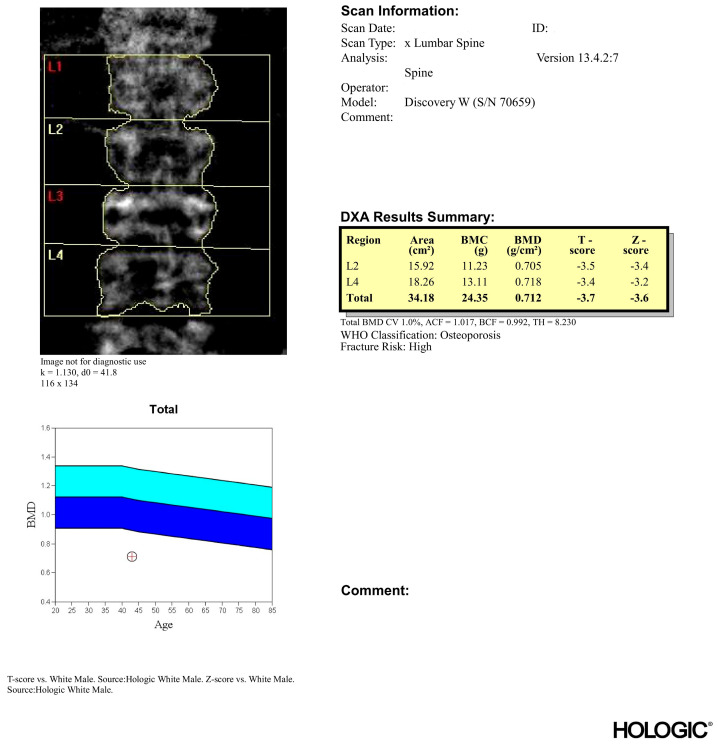

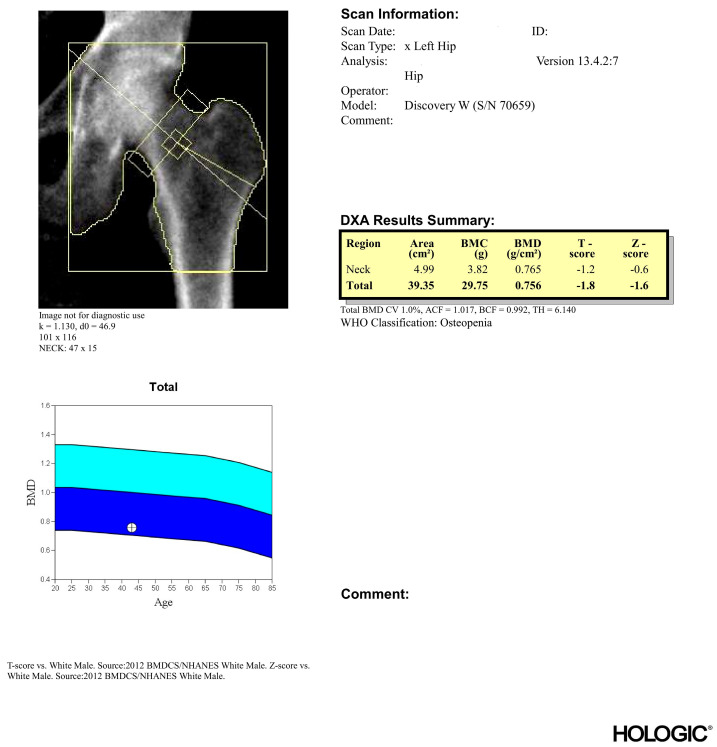
DXA report of case 3 patient, showing reduced T-score.

DXA showed osteoporosis: lumbar spine T-score −3.7 (BMD 0.712 g/cm^2^); femoral neck T-score −1.2 (BMD 0.765 g/cm^2^); and total hip T-score −1.8 (BMD 0.756 g/cm^2^), as shown in Figure 1. Biochemistry showed vitamin D insufficiency (67 nmol/L) with normal calcium, phosphate and renal function. Bone turnover markers were in the mid-normal range (CTX 0.162 µg/L; PINP 34.9 µg/L). Common secondary causes were excluded (primary hyperparathyroidism, hypogonadism, Cushing syndrome, and coeliac disease). The history of severe Hymenoptera anaphylaxis prompted measurement of basal serum tryptase, which was strikingly elevated at 87.0 µg/L (RR < 11.4 µg/L).

BM biopsy demonstrated multifocal clusters of atypical MCs with aberrant CD2/CD25 expression and confirmed the *KIT* D816V mutation. There were no cutaneous lesions. The basal tryptase of 87 µg/L remained below the B-finding threshold of 200 µg/L, and no C-findings were identified. A diagnosis of ISM was established.

Treatment with intravenous zoledronic acid was initiated, and Hymenoptera venom immunotherapy was continued. On follow-up, no additional fractures were recorded. Repeat basal serum tryptase was stable. Given the high MC burden (basal tryptase 87 µg/L) and prior grade IV anaphylaxis, KIT-directed therapy with avapritinib is under multidisciplinary consideration.

### 2.4. Case 4

A 71-year-old woman had osteoporosis and a T11 vertebral fracture attributed to primary hyperparathyroidism, for which she had been receiving regular denosumab for 4 years. Despite ongoing antiresorptive therapy, she sustained multiple vertebral compression fractures two years before the present assessment, presenting with severe back pain and impaired mobility. Comorbidities included nephrolithiasis, rheumatoid arthritis (not currently treated with glucocorticoids), arterial hypertension and chronic obstructive pulmonary disease.

DXA was not performed at the time of the acute presentation; vertebral morphometry demonstrated compression fractures at T2, T8 and T10 with approximately 25% height loss, and 33–50% height loss affecting all lumbar vertebrae. DXA showed femoral neck T-score −3.2 (BMD 0.498 g/cm^2^) and total hip T-score −2.4 (BMD 0.654 g/cm^2^); lumbar spine DXA was not interpretable owing to vertebral fractures and degenerative change. Laboratory testing showed elevated basal serum tryptase 46.1 µg/L (RR < 11.4 µg/L), hypercalcaemia (corrected calcium 2.73 mmol/L; RR 2.10–2.60 mmol/L), vitamin D insufficiency, and a frankly elevated CTX (0.903 µg/L; PINP 49.4 µg/L) consistent with active bone resorption despite prior denosumab.

BM biopsy confirmed ISM: multifocal aggregates of atypical MCs with aberrant CD2/CD25 expression and the *KIT* D816V mutation; no cutaneous lesions; no B- or C-findings attributable to the MC clone. Primary hyperparathyroidism was identified as a coexisting contributor to bone loss.

Antiresorptive therapy was switched from denosumab to intravenous zoledronic acid 5 mg, timed to the end of the denosumab dosing interval to mitigate rebound bone loss. On follow-up, no further fragility fractures were recorded; repeat DXA measurements were stable.

## 3. Discussion

The present cases illustrate the breadth of bone pathology encountered in ISM: all four patients had substantial bone loss despite markedly different clinical presentations. This variability is consistent with prior series reporting osteoporosis in up to 60% of adults with SM and fragility fractures in roughly 40% [2,6,7].

### 3.1. Mechanisms of Bone Loss in ISM

Bone involvement in ISM is driven by dysregulation of the cellular and molecular networks that govern osteoclast-mediated resorption and osteoblast-mediated formation, most notably the canonical WNT pathway and the receptor activator of nuclear factor κB ligand (RANKL)–osteoprotegerin (OPG) axis [3,13,14]. WNT signalling stabilises β-catenin in osteoblast precursors, promoting their differentiation and activity, while the RANKL/RANK/OPG axis regulates osteoclast maturation, with OPG acting as a decoy receptor for RANKL [13,14,15]. In ISM, MC-derived mediators, including histamine, tryptase, heparin and pro-inflammatory cytokines, tip this balance toward resorption by up-regulating RANKL and down-regulating OPG, and by suppressing WNT-dependent osteoblast activity [3,13,14,16]. Direct infiltration of BM by neoplastic MCs further contributes to focal skeletal abnormalities [2].

In our cohort, all four patients harboured the *KIT* D816V mutation, a constitutively activating alteration of the KIT receptor tyrosine kinase that drives MC proliferation independently of stem cell factor and is found in up to 95% of patients with SM [3,5,11,17,18]. This mutation increases the overall MC burden, yielding higher basal tryptase levels while also increasing constitutive and periodic mediator release, leading to typical CNS, GIT, skin and, in some cases, severe skeletal involvement (see Table 2) [2,3,11,19,20].

Case 3, who had the highest tryptase level (87 µg/L) and multiple spontaneous fractures, exemplifies this high-burden phenotype.

Case 2 does not conform to ISM: the co-existence of a clonal non-mast-cell haematological neoplasm (*JAK2* V617F-positive post-ET myelofibrosis) with bone-marrow-confirmed SM places him in the advanced-SM category of systemic mastocytosis with an associated haematological neoplasm (SM-AHN) under the WHO 5th edition (2022) and ICC 2022 classifications. This subtype illustrates a truly multifactorial skeletal pathology in which both the myeloproliferative and the MC clones contribute to bone loss, and a history of repeated low-trauma fractures predated the SM-AHN diagnosis.

SM-AHN encompasses a diverse group of diseases. Frequently, as in our patient, the MC clone is identified only after the diagnosis of the associated haematological neoplasm (AHN) component, or it may be completely overlooked in patients with chronic myeloproliferative neoplasms, especially if no clinical signs of MC mediator release, such as anaphylaxis or osteoporosis, are present [4,19,21,22]. The prognosis in SM-AHN is generally dictated by the type of AHN. In patients with concomitant acute myeloid leukaemia (AML) or chronic myelomonocytic leukaemia (CMML), both prognosis and treatment are primarily driven by the AHN component [17,19,22]. Only in patients who exhibit features of concomitant aggressive mastocytosis with organ damage attributable to MC infiltration is targeted therapy against the MC clone indicated [17,19,22].

In our patient, the disease requiring treatment was the AHN component, specifically post-ET myelofibrosis. We were not able to determine whether the KIT mutation was present in both disease clones; however, in the presence of a *JAK2* mutation, ruxolitinib was therefore the preferred treatment option for the AHN. The concomitant MC clone, reflected by a moderately elevated tryptase level of 43.7 µg/L and *KIT* D816V variant allele frequency (VAF 0.391%), demonstrated indolent clinical behaviour without signs of MC mediator release apart from osteoporosis. We included this case because the MC component behaved indolently and contributed to the clinical manifestations (osteoporosis), making it relevant to the spectrum of ISM presentations despite the presence of an AHN.

Taken together with the broader literature, these observations are consistent with an approach in which in adults with unexplained osteoporosis or treatment-refractory bone loss, molecular screening for *KIT* D816V in peripheral blood by allele-specific or droplet-digital PCR (analytical sensitivity ≈ 10^−4^–10^−5^) may be considered alongside basal serum tryptase, particularly when clinical suspicion is strong, given the higher specificity of the mutation for clonal MC disease than of tryptase elevation alone [20,21]. A positive peripheral-blood KIT D816V result fulfils the molecular minor criterion of SM and can support direct referral to BM evaluation; a negative result does not exclude marrow-restricted disease, and in its presence, a low threshold for diagnostic BM biopsy with immunophenotyping and sensitive KIT sequencing seems prudent, particularly when prior severe anaphylaxis, B-findings, MC-mediator symptoms or fractures on antiresorptive therapy are present. The relative diagnostic yield of this approach in unselected osteoporosis cohorts has not been formally established and warrants prospective evaluation.

### 3.2. Serum Tryptase and Bone Turnover Markers

All four patients had markedly elevated basal serum tryptase (41.4–87.0 µg/L; RR < 11.4 µg/L), reflecting increased MC burden. Bone resorption was heterogeneous: C-terminal telopeptide of type I collagen (CTX) was within the mid-normal range in Cases 1–3, whereas only Case 4 had frankly elevated CTX (0.903 µg/L), consistent with the clinical picture of active bone loss despite prior denosumab therapy. This mixed pattern aligns with previous reports that basal tryptase correlates with MC burden but does not directly predict fracture risk, and that elevated CTX and bone-specific alkaline phosphatase (bALP) have been associated with higher fracture risk in ISM [2,3,5,8,23]. In clinical practice, bone turnover markers can therefore be incorporated alongside, rather than replace, DXA, vertebral imaging and MC-specific work-up when estimating fracture risk in ISM [5,23].

Beyond histamine and tryptase, neoplastic MCs release tumour necrosis factor-α (TNF-α), interleukin-1 (IL-1) and interleukin-6 (IL-6), which enhance osteoclast formation and activity [3,8,13]. IL-1 and TNF-α amplify osteoblast RANKL production and osteoclast-precursor RANK expression, generating a positive feedback loop that progressively shifts bone remodelling toward resorption [3,13]. IL-6 directly activates osteoclasts and promotes their recruitment to bone surfaces [3,8]. Heparin, a major MC-granule constituent, further stimulates osteoclastic resorption, partly by interfering with OPG–RANKL interaction [24,25]. The resulting mediator milieu differs fundamentally from that of postmenopausal osteoporosis, which is primarily driven by oestrogen deficiency [26]. Recent work has identified a complementary mechanism: MC-derived extracellular vesicles carry microRNAs such as miR-23a and miR-30a that target key osteoblast transcription factors, helping explain why some ISM patients lose BMD despite normal or elevated bALP [3,27].

### 3.3. Diagnostic Pitfalls

In all four patients, elevated basal tryptase prompted BM biopsy and subsequent diagnosis. However, serum tryptase is not specific for ISM: it can be raised in hereditary α-tryptasaemia (HαT), severe allergic reactions, obesity, chronic kidney disease and haematological malignancies [28]. A persistently elevated basal value is therefore interpreted in clinical context, and mastocytosis may be considered, potentially leading to BM biopsy with immunophenotyping and KIT mutation analysis [2,3,23]. DXA remains the cornerstone of skeletal assessment, supplemented by vertebral imaging, bone turnover markers and basal tryptase [3]; however, fracture risk prediction is challenging because of altered bone microarchitecture and focal lesions, and low-trauma fractures can occur despite normal or only modestly reduced BMD [10,23,29].

A striking finding in our series is that three of four patients were initially labelled as having idiopathic osteoporosis, with ISM recognised only after basal tryptase was measured. This pattern highlights gaps in the systematic work-up of secondary osteoporosis. Case 3, in particular, experienced grade IV anaphylaxis after a wasp sting, a classic indicator of increased MC burden that would typically support consideration of earlier tryptase and peripheral blood *KIT* D816V testing. The absence of cutaneous lesions in all four patients added to the diagnostic challenge, in keeping with the 10–20% of SM patients in whom skin involvement is lacking and in whom bone disease or anaphylaxis may be the dominant presentation [2,23].

**Figure 2:**
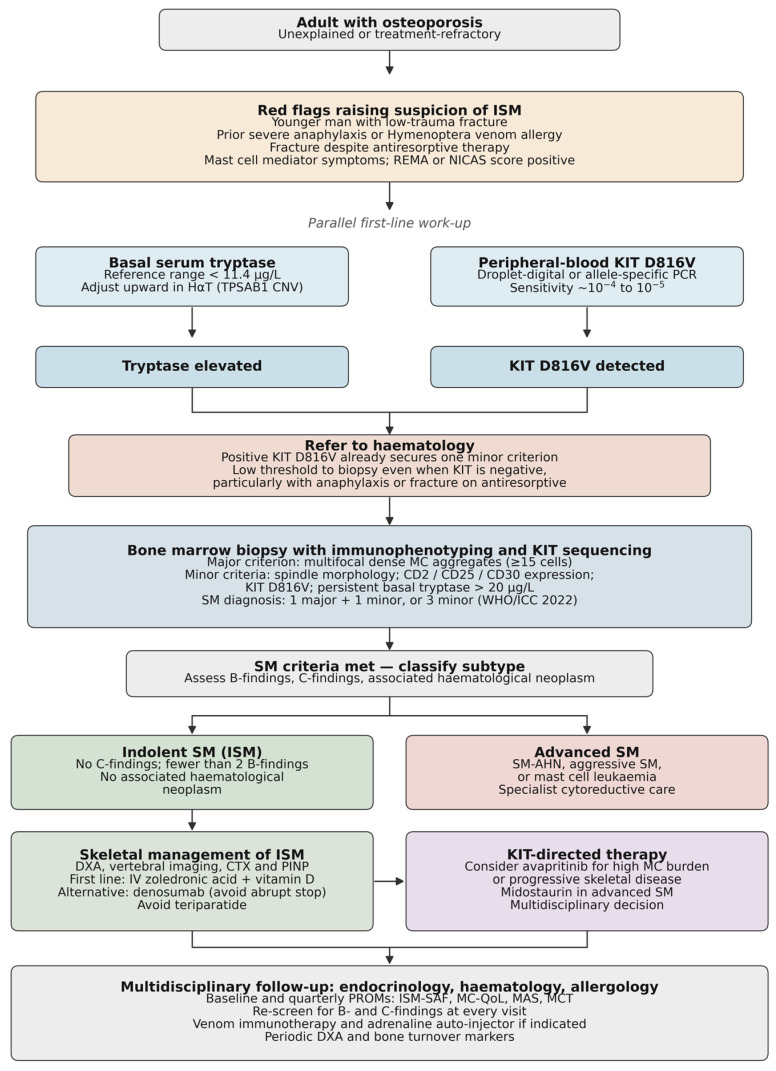
Algorithm for the diagnosis and management of indolent systemic mastocytosis in adults with osteoporosis. Abbreviations: ISM, indolent systemic mastocytosis; SM, systemic mastocytosis; SM-AHN, systemic mastocytosis with an associated haematologic neoplasm; MCs, mast cells; DXA, dual-energy X-ray absorptiometry; CTX, C-terminal telopeptide of type I collagen; PINP, procollagen type I N-terminal propeptide; REMA, the Spanish Network on Mastocytosis; NICAS, national institutes of health idiopathic clonal anaphylaxis score; PROMs, patient-reported outcome measures; ISM-SAF, indolent systemic mastocytosis symptom assessment form; MC-QoL, mastocytosis quality of life questionnaire; MAS, mastocytosis activity score; MCT, mastocytosis control test; HαT, hereditary alpha-tryptasemia; PCR, polymerase chain reaction.

Our observations are in line with prior reports suggesting that incorporating basal serum tryptase into the evaluation of unexplained osteoporosis—particularly in younger men, in patients with prior severe anaphylaxis and in those who fracture on antiresorptive therapy—may be a low-cost and informative step [2,3]; the actual diagnostic yield in routine practice should ideally be quantified in larger, prospective cohorts. The integrated screening and management pathway derived from these considerations is presented in Figure 2.

### 3.4. Assessment of Systemic Mastocytosis

Diagnosis of SM, including ISM, follows the 2022 WHO/ICC criteria. The diagnosis of SM requires the major criterion plus ≥1 minor criterion, or ≥3 minor criteria. The major criterion is the presence of multifocal dense MC infiltrates (≥15 cells per aggregate) in BM or another extracutaneous organ. Minor criteria include atypical/spindle MC morphology, *KIT* D816V or related codon-816 mutations, aberrant CD2/CD25/CD30 expression, and basal serum tryptase > 20 µg/L (with adjustment in hereditary α-tryptasaemia) [17,19,22].

Classification as ISM requires the absence of B-findings (markers of high MC burden such as >30% BM infiltration, tryptase > 200 µg/L, *KIT* D816 VAF > 10%, organomegaly without dysfunction, or dysmyelopoiesis) and absence of C-findings (organ damage attributable to MC infiltration, including cytopenias, hepatic dysfunction/ascites, hypersplenism, malabsorption with weight loss, or large osteolytic lesions with fractures). These must be reassessed at diagnosis and each follow-up, as their emergence mandates risk re-stratification and may indicate the need for cytoreductive therapy [19,30]. In our cohort, no patient fulfilled B- or C-findings.

Because ISM morbidity extends beyond skeletal disease, standardised assessment of mediator-related symptoms is increasingly recommended. Validated patient-reported outcome measures (PROMs) include the ISM-SAF, MC-QoL, MAS, MSSDD, MCT, and generic tools such as BPI-SF and EQ-5D-5L [31,32,33,34]. Although not applied in our cases, their systematic use enables quantification of symptom burden not captured by tryptase or BMD, supports earlier detection of progression, and informs patient-centred decisions regarding symptomatic, antiresorptive, or KIT-directed therapy [19,30].

Because ISM morbidity extends well beyond the skeleton, objective quantification of MC-mediator symptom burden using validated patient-reported outcome measures (PROMs) is increasingly recommended as part of comprehensive ISM care and, where feasible, can be incorporated into the diagnostic and monitoring work-up alongside tryptase, KIT testing, DXA and bone-turnover markers [30]. We did not use these instruments in the present cases and therefore make no claims about their performance in our cohort; we refer to them here only to flag a gap in our own practice that future work should address.

### 3.5. Therapeutic Considerations

Management of ISM-related bone disease requires a mechanism-based approach that addresses both ongoing bone loss and underlying MC proliferation. Bisphosphonates—in particular intravenous zoledronic acid—constitute the first-line antiresorptive choice. Through inhibition of farnesyl pyrophosphate synthase in the mevalonate pathway, they disrupt prenylation of small GTPases critical for osteoclast function and survival and act independently of the dysregulated RANKL/OPG axis characteristic of ISM [7,11,35,36,37]. Annual intravenous zoledronic acid has been shown to increase BMD at the lumbar spine and hip, reduce bone turnover markers and lower fracture risk in patients with mastocytosis-related osteoporosis [2,5,11,38]. In line with these data, all four of our patients were treated or transitioned to zoledronic acid; denosumab, which inhibits osteoclast formation upstream by blocking the RANKL–RANK interaction, is a reasonable alternative and has been reported to both improve BMD and to reduce serum tryptase, although the evidence base remains small [3,12,38]. Because abrupt discontinuation of denosumab is associated with rebound bone loss, the switch in Case 4 was planned carefully, with zoledronic acid timed to the end of the denosumab dosing interval.

Teriparatide, an osteoanabolic parathyroid-hormone analogue, may warrant caution in ISM, given that parathyroid-hormone signalling has been shown to stimulate MC degranulation and survival, potentially increasing histamine and tryptase release and further perturbing the RANKL/OPG axis [3,26,27,28,39]. In our practice, teriparatide was not used in Case 1 after ISM was confirmed and was avoided in Case 3 because of severe skeletal disease coupled with a very high MC burden.

Antiresorptive therapy targets downstream bone resorption but does not address MC proliferation or mediator release. KIT-targeted tyrosine-kinase inhibitors, midostaurin and, more selectively, avapritinib, reduce MC burden and systemic symptoms [3,19,40,41]. Avapritinib inhibits *KIT* D816V; curbs autonomous MC growth and reduces histamine, tryptase and pro-inflammatory cytokine production [3,19,41]. In the PIONEER trial it produced meaningful symptom reduction and substantial normalisation of serum tryptase in ISM [19,41]. Emerging data specifically addressing the skeleton are encouraging: avapritinib-treated ISM patients have shown BMD increases at the lumbar spine, total hip and femoral neck at three years (with measurable gains already at one year), normalisation of tartrate-resistant acid phosphatase 5b as early as 24 weeks (a pattern unaltered by concurrent antiresorptive therapy) and reductions in procollagen type I N-terminal propeptide [42].

For ISM patients with severe skeletal involvement and high MC burden, such as the phenotype exemplified by Case 3, the combination of antiresorptive therapy with KIT-directed treatment is a biologically plausible strategy that addresses both bone loss and MC-mediated disease activity; the actual incremental benefit on skeletal outcomes, however, requires evaluation in dedicated prospective studies before this combination can be generally recommended.

## 4. Limitations

Several limitations warrant mention. This is a small, single-centre retrospective series and is subject to both selection and referral bias, as all four patients were identified through a tertiary metabolic bone unit; milder or asymptomatic phenotypes are therefore under-represented. The observed case characteristics may not reflect the broader ISM population presenting with osteoporosis. The series does not allow definitive inferences about screening strategy or treatment effectiveness. The absence of a control group and the small sample size preclude formal causal inference about the contribution of MC mediators versus coexisting factors (e.g., *JAK2*-positive myelofibrosis in Case 2 and primary hyperparathyroidism in Case 4) to the observed bone loss. DXA was not available at the time of diagnosis in Case 4, limiting quantitative comparison. Finally, the follow-up is short relative to the natural history of ISM and does not allow conclusions about long-term skeletal outcomes. Validated patient-reported outcome measures were not applied, leaving symptom burden incompletely characterised. Finally, follow-up is short relative to the natural history of ISM and does not permit conclusions about long-term skeletal outcomes, fracture incidence or the durability of treatment response.

The cases are therefore presented as illustrative of the diagnostic pathway from unexplained osteoporosis to recognition of systemic mastocytosis, rather than as a basis for general recommendations on screening or treatment, which require evaluation in larger prospective cohorts.

## 5. Conclusions

In this small retrospective series, four adults referred to a tertiary metabolic-bone unit for unexplained or treatment-refractory osteoporosis were diagnosed with systemic mastocytosis after basal serum tryptase, measured during the secondary-osteoporosis work-up, was found to be elevated and prompted bone-marrow biopsy with *KIT* D816V analysis. Three patients (Cases 1, 3 and 4) fulfilled the WHO 2022 criteria for ISM; cutaneous involvement was absent in all four. The fourth (Case 2), with established *JAK2* V617F-positive post-essential-thrombocythaemia myelofibrosis, was classified as SM with associated haematological neoplasm (SM-AHN); his MC clone, characterised by a moderately elevated tryptase (43.7 µg/L) and a low *KIT* D816V variant allele frequency (0.391%), behaved indolently and contributed clinically through osteoporosis alone—illustrating how an indolent MC component can coexist with, and be obscured by, a chronic myeloid neoplasm.

Several recurring observations emerge from these four patients: osteoporosis or a low-trauma fracture can be the only presenting feature of SM; the absence of skin lesions does not exclude the diagnosis; basal serum tryptase, measured as part of a structured work-up of secondary osteoporosis, was in each case the step that led to diagnosis; and management was inherently multidisciplinary, spanning endocrinology, haematology and, where relevant, allergology. All four patients received intravenous zoledronic acid with vitamin D repletion, in line with the current literature; KIT-targeted therapy was considered but not initiated, and follow-up is too short to comment on long-term skeletal outcomes.

A four-patient retrospective series cannot define screening pathways, treatment efficacy or long-term outcomes, and the broader diagnostic and therapeutic considerations discussed above are drawn from the current WHO/ICC/AIM-ECNM 2022–2024 guidance and from published cohorts rather than from our own data. Larger prospective studies are needed for better screening and treatment practices for osteoporosis-presenting ISM.

## Figures and Tables

**Table 1:** Clinical, laboratory, densitometric and radiological findings of the four patients with systemic mastocytosis (Cases 1, 3 and 4: ISM; Case 2: SM-AHN with JAK2-positive post-ET myelofibrosis).

Parameter	Case 1	Case 2	Case 3	Case 4
Sex/Age (years)	Male/55	Male/39	Male/43	Female/71
Fracture sites	L5 vertebra	Left radius and ulna (age 15, refracture at 26); T12 and L1 vertebrae	T12 and L3 vertebrae; rib fracture	T2, T8, T10 vertebral compression fractures; height loss of T11, T12 and all lumbar vertebrae
Mutation	*KIT* D816V	*KIT* D816V *JAK2* V617F (post-ET myelofibrosis)	*KIT* D816V	*KIT* D816V
WHO 2022 classification	ISM	SM-AHN § (SM + post-ET MF)	ISM	ISM
Serum tryptase (µg/L) *	41.4	43.7	87.0	46.1
CTX (µg/L) †	0.092	0.274	0.162	0.903
PINP (µg/L) ‡	67.5	50.9	34.9	49.4
Lumbar spine T-score (SDS)	−4.0	−2.5	−3.7	Not available
Lumbar spine BMD (g/cm^2^)	0.612	0.843	0.712	Not available
Femoral neck T-score (SDS)	−2.1	−1.4	−1.2	−3.2
Femoral neck BMD (g/cm^2^)	0.650	0.733	0.765	0.498
Total hip T-score (SDS)	−1.3	−0.6	−1.8	−2.4
Total hip BMD (g/cm^2^)	0.844	0.941	0.756	0.654
Bone-directed therapy	Zoledronic acid	Zoledronic acid; ruxolitinib; warfarin	Zoledronic acid; venom immunotherapy	Denosumab → zoledronic acid
Prior anaphylaxis	No	No	Yes (grade IV, Hymenoptera sting)	No
Key comorbidities/risk factors	Vitamin D deficiency; maternal osteoporosis	JAK2-positive myelofibrosis; splanchnic vein thrombosis	Former smoker	Primary hyperparathyroidism; rheumatoid arthritis; hypertension; COPD

* Reference range for basal serum tryptase: <11.4 µg/L. † CTX reference range: 0.142–1.351 µg/L. ‡ PINP reference range: 27.7–127.6 µg/L. Abbreviations: BMD, bone mineral density; COPD, chronic obstructive pulmonary disease; CTX, C-terminal telopeptide of type I collagen; ET, essential thrombocythemia; ISM, indolent systemic mastocytosis; PINP, procollagen type I N-terminal propeptide; SM, systemic mastocytosis. § Case 2 was classified as SM-AHN (systemic mastocytosis with associated haematological neoplasm) under the WHO 5th edition (2022) and ICC 2022 criteria, an advanced-SM category distinct from ISM and associated with a less favourable prognosis. The mast cell clone in this patient behaved indolently and contributed clinically through osteoporosis alone.

**Table 2:** MC degranulation symptoms by organ system (adapted from the 2026 National Comprehensive Cancer Network Guideline on Systemic Mastocytosis).

Systemic	Skin	Gastrointestinal	CNS	Osteomuscular
Anaphylaxis; fatigue; lightheadedness	Flushing (face, neck, chest); pruritus; urticaria; angioedema; dermatographism; urticaria pigmentosa	Diarrhoea; nausea; vomiting; abdominal pain; bloating; gastric distress; gastroesophageal reflux disease; malabsorption	Headache; cognitive dysfunction; difficulty concentrating; memory problems; anxiety; depression; fatigue	Bone pain; muscle pain; osteoporosis; osteopenia; osteosclerosis; pathologic fractures

## Data Availability

The data supporting the findings of this case series are available from the corresponding author upon reasonable request. Data are not publicly available because of patient privacy restrictions.
